# Hiatal Hernia With Gastric Perforation

**DOI:** 10.7759/cureus.16572

**Published:** 2021-07-22

**Authors:** Dieter Brummund, Angela Chang, Michael Renda

**Affiliations:** 1 Department of General Surgery, Aventura Hospital and Medical Center, Aventura, USA; 2 Department of Anesthesiology, Aventura Hospital and Medical Center, Miami, USA; 3 Department of General Surgery, Kendall Regional Medical Center, Miami, USA

**Keywords:** hiatal hernia, acute abdomen, cameron ulcer, marginal ulcer, gastric ulcer, gastric perforation, perforated viscus, pneumoperitoneum

## Abstract

A 63-year-old male with a history of hiatal hernia presented with one day of hematemesis and acute peritonitis. Computed tomographic imaging revealed perigastric pneumoperitoneum concerning perforated viscus. Exploratory laparotomy revealed a Type III hiatal hernia with a perforated posterior gastric ulcer, which was reduced and repaired. This report describes a case of acute abdomen secondary to hiatal hernia, a rare presentation of hiatal hernia, along with its surgical management and postoperative care.

## Introduction

A hiatal hernia is a laxity of the esophageal hiatus and phrenoesophageal membrane wherein the intra-abdominal contents, most commonly the gastric cardia, herniates into the thorax. Hiatal hernia is classified into four types according to the location and degree of herniation. The most common is the Type I sliding hiatal hernia, in which the gastroesophageal junction (GEJ) and esophageal hiatus herniate into the thorax. Type II paraesophageal hernias involve a portion of the stomach herniating through the esophageal hiatus while the GEJ remains in its native position. A Type III hernia involves both the esophageal hiatus and a portion of the stomach, whereas a Type IV hiatal hernia involves any portion of the stomach with additional abdominal viscera [1].

Hiatal hernias are often asymptomatic. If symptomatic, symptoms vary according to type and reflect the underlying pathophysiology. Type I hiatal hernias are associated with gastroesophageal reflux disease and are related to the displacement of the esophageal-gastric junction into the chest and loss of lower esophageal sphincter resting tone. Types II-IV hiatal hernias present with pain, nausea, fullness, or vomiting, which are associated with ischemia and obstruction due to rotation of the stomach or a herniated segment on its axis. As the stomach is still fixed at the GE junction, the greater curvature migrates up into the thorax, causing the stomach to rotate around its longitudinal axis, leading to gastric volvulus, obstruction, incarceration, perforation, and other acute and potentially catastrophic pathology [1]. Hiatal hernias can additionally present with upper gastrointestinal bleeding secondary to gastric ulcerations at the level of herniation, known as Cameron ulcers. These ulcers are attributed to impingement of the stomach at the level of herniation and results in mucosal ischemia [2,3]. Asymptomatic hiatal and paraesophageal hernias become symptomatic and necessitate repair at a rate of 1% per year [4,5]. While traditionally managed on an elective basis if asymptomatic, contemporary studies have suggested non-inferiority and increased quality of life with a watchful waiting approach [6].

This case report describes a unique case of a hiatal hernia presenting as an acute abdomen secondary to gastric perforation with a subsequent successful repair.

## Case presentation

**Figure 1:**
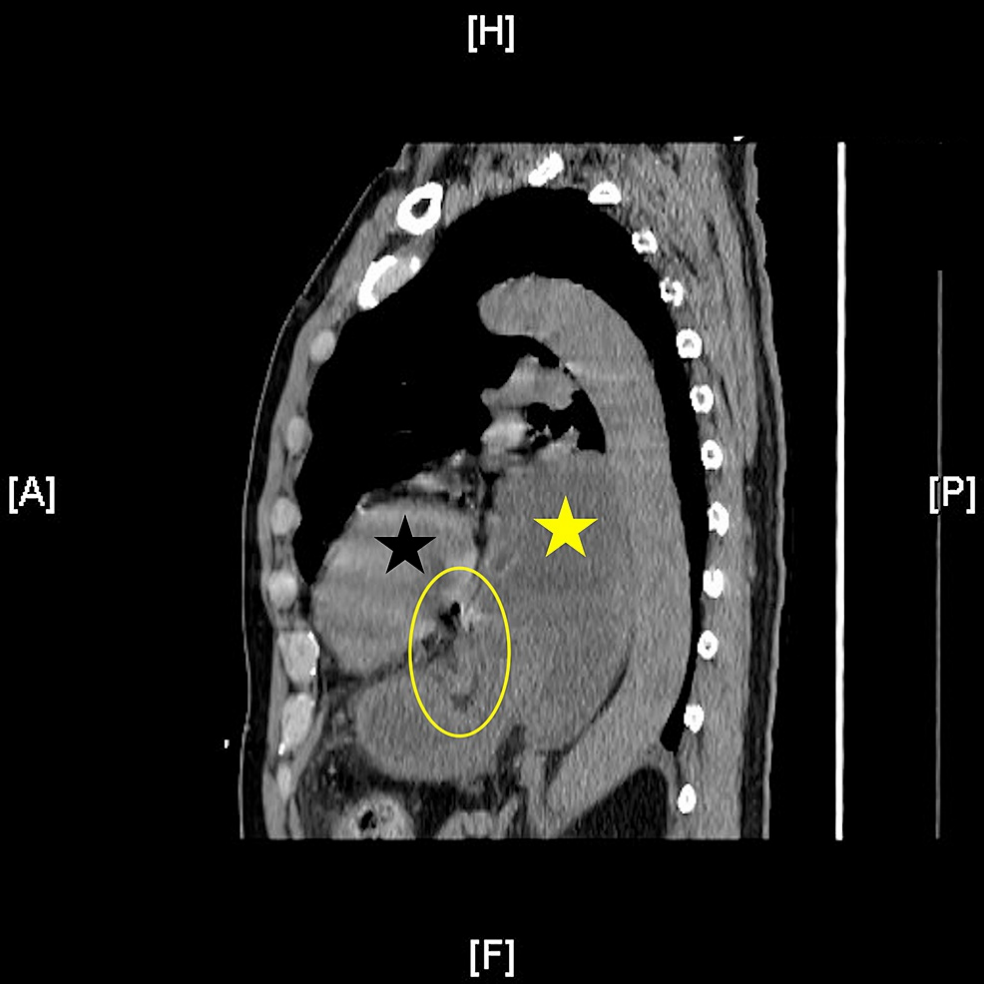
Type III hiatal hernia sagittal view showing gastric wall edema, gastric pneumatosis, and punctate pneumoperitoneum Note heart (solid black star) and retrocardiac position of stomach (solid yellow star) with the majority of the stomach above the diaphragm consistent with Type III hiatal hernia. Note thickening of gastric wall, focal gastric pneumatosis, and punctate pneumoperitoneum (transparent yellow oval) suspicious of ischemia and gastric perforation.

**Figure 2:**
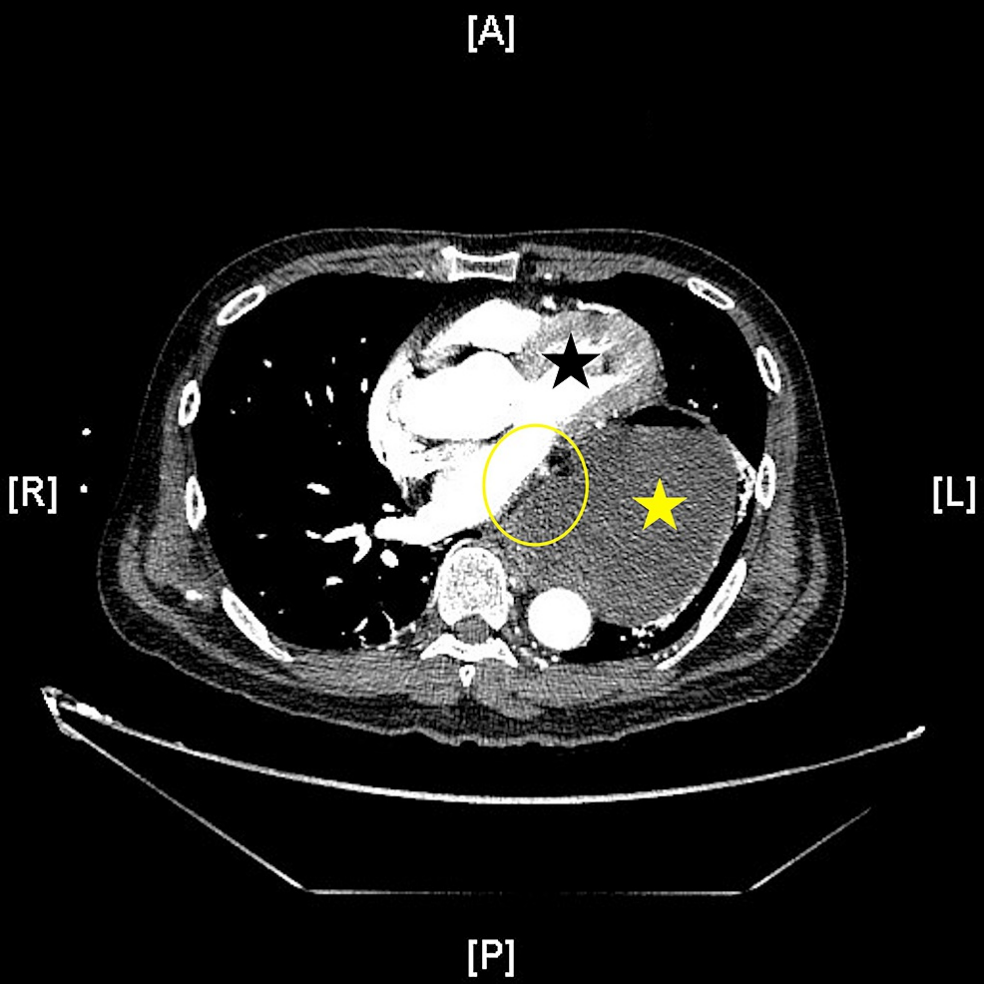
Type III hiatal hernia transverse view showing punctate pneumoperitoneum and gastric pneumatosis Note heart (solid black star) and retrocardiac position of stomach (solid yellow star) consistent with Type III hiatal hernia. Note punctate pneumoperitoneum and pneumatosis in the gastric wall immediately posterior to the heart (transparent yellow oval) suggestive of gastric perforation.

A 63-year-old male presented with a one-day history of diffuse abdominal pain, nausea, hematemesis, and bright red blood per rectum. He reported a past medical history significant for gastroesophageal reflux disease. Family history was significant for gastric cancer in the patient's father at age 67 and colon cancer in his brother at age 55. On physical examination, the patient appeared toxic and was hypotensive and tachycardic with a fever of 38℃. He had abdominal distention as well as diffuse tenderness and guarding, characteristic of peritonitis. Laboratory investigations revealed leukocytosis of 23,800 cells/µL (reference range: 4,500-11,000 cells/µL), polycythemia with a hemoglobin of 17.8 g/dL (reference range: 13.7-17.5 g/dL), an elevated anion gap of 22 (reference range: 10-20), lactic acidosis of 5 mmol/L (reference range: 0.4-2 mmol/L), pH of 7.23 (reference range: 7.35-7.45), and a base deficit of 10 (reference range: -2 to 2). Computed tomographic (CT) imaging of the chest, abdomen, and pelvis was done, given a high index of suspicion for esophageal perforation or perforated viscus from the clinical history of hematemesis and the physical exam consistent with peritonitis. CT revealed a large distended and fluid-filled hiatal hernia containing the stomach with punctate pneumoperitoneum near the gastric antrum and diffuse fat stranding of the omentum (Figures 1, 2).

Given the concern for intra-abdominal catastrophe in a patient with hemodynamic instability and peritonitis, the decision was made to emergently take the patient to the operating theater. A midline laparotomy incision was made from the xiphoid to below the umbilicus. Upon entering the peritoneum, murky black fluid was encountered. The abdominal contents were inspected, and a large Type III hiatal hernia was found containing the majority of the stomach. No torsion or volvulus was present. The hernia was reduced, and the stomach was brought into the abdominal cavity with no perforation noted to the anterior gastric wall, but an even greater volume of murky black fluid was then encountered emanating from the hernia sac. At this point, the lesser sac was entered, and the stomach was mobilized off the transverse colon and reflected, revealing a perforated ulcer on the posterior wall of the stomach in direct proximity to where it is laid on the hernia margin, consistent with Cameron ulcer. The gastric perforation was biopsied and repaired with both layered gastrorrhaphy and an omental graham patch. The hiatal hernia was not repaired given the contaminated field, with the decision made for the patient to return at a later date to address it. Two intra-abdominal Jackson-Pratt drains were placed, the fascia was closed, and a vacuum-assisted closure device was placed over the surgical wound. Additional medical management was instituted, including intravenous antibiotics and antifungals given the proximal location of the perforation, intravenous pantoprazole, nothing per os, and a nasogastric tube on low intermittent suction for gastric decompression.

Postoperatively, the patient improved and was extubated on postoperative day 1. On postoperative day 4, an esophagram and upper gastrointestinal tract radiography were obtained, which showed gastric reflux extending to the proximal third of the esophagus with no active extravasation. At the same time, the patient also underwent delayed primary closure of the laparotomy wound at the bedside. The patient was then started on a clear liquid diet, which was well-tolerated and advanced to a soft diet on postoperative day 7. The gastric ulcer biopsy was negative for *Helicobacter pylori* or malignancy. The patient continued to improve, and he was discharged home on postoperative day 8.

## Discussion

In the acute setting, gastric perforation, incarceration, or volvulus due to hiatal hernias is a rare cause of acute abdomen. This presentation is associated with a high degree of morbidity and mortality, and emergent operative intervention is required. A 10-year retrospective study of 37 patients in the United Kingdom by Bujoreanu et al. found a high rate of morbidity and mortality with the emergent repair of giant hiatal hernias. Approximately 35% of patients developed pneumonia, 24.3% developed end-organ dysfunction requiring intensive care, 5.4% underwent revision surgery, and 5.4% died within 30 days [7].

With regard to gastric perforation within a hiatal hernia, a review of the literature was performed using the PubMed database. A search was performed for studies in the English language performed within the last 20 years. Five case reports describing gastric perforation within a hiatal hernia were found [8-13], of which all five underwent emergency laparotomy. The mean age was 74.2 years (range: 61-91 years). There was a mortality rate of 20% with one patient dying on postoperative day 2. The average length of stay was 40.75 days for the surviving patients (range: 10-92 days) with a shorter stay correlating with younger age and fewer comorbidities (Table 1).

**Table 1:** A literature review of gastric perforation within hiatal hernia

Study	Age	Gender	Hernia Type	Length of Stay	Repair	Complication
Maruyama et al., 2001 [13]	71	Female	Type III	40 days	Wedge resection, Graham patch	Respiratory failure
Pol et al., 2008 [8]	61	Male	Type II	10 days	Primary repair, Graham patch	Intrathoracic abscess
Parker et al., 2011 [10]	72	Female	Type I	21 days	Distal esophagectomy with gastric pull-up	Vagus nerve injury
Fukai et al., 2019 [11]	91	Female	Type III	Expired	Partial gastrectomy with Roux-en-Y	Death
Wang et al., 2019 [9]	76	Male	Type IV	92	Total gastrectomy with Roux-en-Y	Anastamotic leak requiring esophagostomy and decortication

Past literature, including a literature review by Meredith et al. in 1980, described up to a 60% mortality rate in patients with hiatal hernia and gastric perforation, with the recommendation for elective surgery as the treatment of choice in patients with hiatal hernia [14]. In the present article, we have found a lower mortality rate, which may reflect an increased speed in diagnosis with newer imaging techniques including computed tomography, improved resuscitation techniques, contemporary critical management, and novel antimicrobials. These advancements and the subsequent improved outcomes underlie contemporary Society of American Gastrointestinal Endoscopic Surgeons (SAGES) guidelines of a watchful waiting approach for asymptomatic hiatal hernia [15].

The patient described in this case recovered and was discharged on postoperative day 8. Unique features leading to a successful outcome included the patient's relatively young age, the relative lack of comorbidities, the very short time from initial evaluation in the emergency department to the operating theater, and focused resuscitative efforts pre- and postoperatively to stabilize the patient and limit secondary end-organ damage from shock.

## Conclusions

This case report describes a unique case of a hiatal hernia complicated by gastric perforation and peritonitis. Though the patient initially presented with upper gastrointestinal bleeding, peritoneal features and hemodynamic instability triggered emergent surgical intervention with laparotomy that revealed a Type III hiatal hernia with a perforated posterior gastric ulcer, which was reduced and repaired successfully. This case report highlights the importance of urgent evaluation and surgical management that can be life-saving in such cases.
